# Partial Restoration of Spinal Cord Neural Continuity *via* Sural Nerve Transplantation Using a Technique of Spinal Cord Fusion

**DOI:** 10.3389/fnins.2022.808983

**Published:** 2022-02-14

**Authors:** Xiaoping Ren, Weihua Zhang, Jian Mo, Jie Qin, Yi Chen, Jie Han, Xinjian Feng, Linxuan Han, Sitan Feng, Haibo Liang, Liangjue Cen, Xiaofei Wu, Chunxing Huang, Haixuan Deng, Zhenbin Cao, Huihui Yao, Rongyu Lan, Xiaogang Wang, Shuai Ren

**Affiliations:** ^1^Department of Orthopedics, Ruikang Hospital Affiliated to Guangxi University of Chinese Medicine, Nanning, China; ^2^Institute of Orthopedics, Ruikang Hospital Affiliated to Guangxi University of Chinese Medicine, Nanning, China; ^3^Global Initiative to Cure Paralysis (GICUP), Columbus, OH, United States; ^4^Department of Imaging, Ruikang Hospital Affiliated to Guangxi University of Chinese Medicine, Nanning, China; ^5^Department of Electrophysiology, Ruikang Hospital Affiliated to Guangxi University of Chinese Medicine, Nanning, China; ^6^Department of Orthopedics, The Second Affiliated Hospital of Harbin Medical University, Harbin, China

**Keywords:** spinal cord injury, spinal cord fusion, clinic trial, polyethylene glycol, paraplegia, GEMINI, cord central pain, spinal cord transplantation

## Abstract

**Background:**

Spinal cord injury (SCI) can cause paralysis and serious chronic morbidity, and there is no effective treatment. Based on our previous experimental results of spinal cord fusion (SCF) in mice, rats, beagles, and monkeys, we developed a surgical protocol of SCF for paraplegic human patients. We designed a novel surgical procedure of SCF, called sural nerve transplantation (SNT), for human patients with lower thoracic SCI and distal cord dysfunction.

**Methods:**

We conducted a clinical trial (ChiCTR2000030788) and performed SNT in 12 fully paraplegic patients due to SCI between T1 and T12. We assessed pre- and postoperative central nerve pain, motor function, sensory function, and autonomic nerve function. Conduction of action potentials across the sural nerve transplant was evaluated. Neural continuity was also examined by diffusion tensor imaging (DTI).

**Results:**

Among the 12 paraplegic patients enrolled in this clinical trial, seven patients demonstrated improved autonomic nerve functions. Seven patients had clinically significant relief of their symptoms of cord central pain. One patient, however, developed postoperative cord central pain (VAS: 4). Five patients had varying degrees of recovered sensory and/or motor functions below the single neurologic level 1 month after surgery. One patient showed recovery of electrophysiologic, motor-evoked potentials 6 months after the operation. At 6 months after surgery, DTI indicated fusion and nerve connections of white cord and sural nerves in seven patients.

**Conclusion:**

SNT was able to fuse the axonal stumps of white cord and sural nerve and at least partially improve the cord central pain in most patients. Although SNT did not restore the spinal cord continuity in white matter in some patients, SNT could restore spinal cord continuity in the cortico-trunco-reticulo-propriospinal pathway, thereby restoring in part some motor and sensory functions. SNT may therefore be a safe, feasible, and effective method to treat paraplegic patients with SCI. Future clinical trials should be performed to optimize the type/technique of nerve transplantation, reduce surgical damage, and minimize postoperative scar formation and adhesion, to avoid postoperative cord central pain.

**Clinical Trial Registration:**

[http://www.chictr.org.cn/showproj.aspx?proj=50526], identifier [ChiCTR2000030788].

## Introduction

Spinal cord injury (SCI) is one of the most devastating diseases in the world. Effective treatment with restoration of sensory and motor function remains one of the greatest challenges in neuroscience. About 27 million people worldwide have been chronically disabled as a result of SCI (James et al., 2019). In addition, there could be 10,000–20,000 new SCI patients in the United States and 60,000 new SCI patients in China every year (Qiu, 2009; National Spinal Cord Injury Statistical Center, 2014). China has the largest number of SCI patients in the world. It is estimated that the direct lifetime cost of care ranged from $1.1 million to $4.7 million per person for more than one million SCI people in North America. For SCI patients in North America alone, the total direct cost of acute treatment and chronic care in the United States exceeded $7 billion per year (Badhiwala et al., 2018). Therefore, it is extremely important to find an effective treatment for SCI.

In recent years, we have proposed and been studying a new concept for SCI treatment called GEMINI spinal cord fusion (SCF) (Canavero, 2015; Canavero and Ren, 2016; Canavero et al., 2016). In this protocol, an extremely sharp surgical knife quickly and relatively atraumatically cuts the spinal cord, resulting in a complete spinal cord transection. Topical application of a fusogen (polyethylene glycol, PEG) can acutely fuse the membranes of the transected axons in the stumps of the transected spinal cord to restore spinal nerve anatomic and electrical continuity across the site of the spinal cord transection (Canavero and Ren, 2016; Canavero et al., 2016, 2017; Ren et al., 2019). To verify the efficacy of SCF, our team initially used rodents as small animal experimental models to carry out SCF research. Studies have shown that mice and rats treated with PEG gradually recovered motor function in their hind limbs. Through the detection of somatosensory evoked potentials (SSEPs) and diffusion tensor imaging (DTI, a novel magnetic resonance imaging technique that assesses the microstructural integrity of nerve fiber tracts; D’Souza et al., 2017), we showed that animals treated with topical PEG had restoration of the conduction of action potentials and the neural continuity at the site of spinal cord transection (Ye et al., 2016; Ren et al., 2017). Based on these findings, our team further validated the efficacy of SCF in large animals (beagles and monkeys) and successfully confirmed the above DTI and electrophysiological results seen in rodents. In addition, beagles and monkeys regained hind leg standing and crawling function at 2 and 3 months postoperatively, respectively. We also verified the role of PEG in restoring nerve continuity *via* histology examinations (Liu et al., 2018; Ren et al., 2019; Ren and Canavero, 2020).

Currently, most paraplegic patients have chronic SCI from the remote trauma. The majority of participants in clinical SCI trials are also patients with chronic SCI. For example, in 2005, a study reported an omental–collagen bridge procedure in a patient with chronic SCI for 3 and one half years (Goldsmith et al., 2005). In 2014, another study reported a technique of bridging the defect in the spinal cord at the site of injury using a peripheral nerve bathed with bulbar olfactory ensheathing cells in a patient with chronic SCI for 21 months (Tabakow et al., 2014). A linearly ordered collagen scaffold reported in 2016 was used to treat five patients with chronic SCI for an average time of 13 months (Xiao et al., 2016). One thing that all three studies have in common with the treatment of SCI is the removal of glial scars in the area of SCI prior to bridging the spinal stumps using different substances. Participants in these three studies also had different degrees of neurological recovery after surgery. Because all the participants were patients with chronic SCI, the postoperative functional recovery time window was longer than our previous animal experiments.

Because our previous animal experiments were performed in an acute spinal cord transection model, in order to transform SCF into the clinic, we needed to transform SCF into the clinical practice; therefore, our team developed several new surgical models for SCF in clinical paraplegic patients and conducted a clinical trial (ChiCTR2000030788)^1^ of SCF. The first model for clinical translational (Model I) was the vascular pedicle hemisected spinal cord transplantation (vSCT). This surgical model consists of removing the area of SCI scar area to produce two acutely transected spinal cord stumps. Half of the spinal cord tissue with the posterior spinal artery was cut from the distal or proximal spinal cord stump to serve as a neural bridge between the distal and proximal spinal cord stumps. In addition, our team conducted preclinical experiments of vSCT in a beagle animal model to verify the feasibility and effectiveness of vSCT. Compared with the control group who showed no recovery of function, beagles treated with local PEG fusion during the vSCT showed recovery of spinal nerve continuity in the postoperative DTI study. In addition, PEG-treated beagles regained some ability to stand and crawl on their hind legs 2 months after the vSCT. The histological examination also demonstrated restored spinal cord continuity in the PEG-treated beagles (Ren et al., 2021). During the subsequent clinical recruitment process, we found that some patients had a SCI in the lower thoracic area or had marked distal spinal cord atrophy (distal cord dysfunction). Cutting half of the spinal cord tissue from the proximal spinal cord tissue site would lead to ascent of the single neurologic level. Therefore, these patients were not suitable for the vSCT treatment. To treat these paraplegic patients, based on a clinical trial of peripheral nerves for SCI reported in 2014 (Tabakow et al., 2014), we developed a second clinical translational model of SCF (Model II), sural nerve transplantation (SNT). In this article, we present the preliminary results of 12 patients with the above-mentioned conditions after SNT treatment to demonstrate the safety, feasibility, and effectiveness of SNT.

## Materials and Methods

### Spinal Cord Fusion Clinical Trial

The clinical trial was approved by the Medical Ethics Committee of Ruikang Hospital Affiliated to Guangxi University of Traditional Chinese Medicine and registered at http://www.chictr.org.cn/index.aspx (ChiCTR2000030788). All treatment operations followed the ethical standards of the hospital and the 1975 Declaration of Helsinki and its subsequent revisions and similar ethical standards. An informed consent form was developed and approved by the Medical Ethics Committee of Ruikang Hospital, affiliated with the Guangxi University of Traditional Chinese Medicine.

### Patients

Inclusion criteria were as follows: complete loss of sensory and motor functions below the injury, a single SCI between T1 and T12, complete spinal cord nerve fiber interruption as confirmed by magnetic resonance imaging (MRI) and diffusion tensor imaging (DTI), complete loss of spinal cord action potential conduction as confirmed by somatosensory evoked potential (SSEP) and motor evoked potential (MEP), under the age of 50 years, satisfactory patient initiative and cooperative spirit, no mental disorders, and normal cardiopulmonary function to complete prolonged operation (ASA:1-2) (Doyle et al., 2021).

### Sural Nerve Transplantation Preparation

Patients completed routine examinations before the surgery, including laboratory examination, electrocardiogram, and ultrasonography. In addition, all patients provided written informed consent. During this process, which was filmed with patient consent, we informed patients of the potential surgical risks, complications, and prognosis for the SCF clinical trial. Patients stopped smoking 2 weeks before the operation. Tilopidine and clopidogrel, aspirin, and non-steroidal anti-inflammatory drugs were discontinued 10, 7, and 2 days before the operation, respectively (None of the patients in the trial were treated with these drugs). Conditions, such as malnutrition, hypertension, and high blood sugar were addressed and controlled before the surgery. Prophylactic antibiotics were administered 30 min before the surgery. Tranexamic acid was administered to minimize bleeding during the surgery.

### Sural Nerve Transplantation Surgical Protocol

**FIGURE 1:**
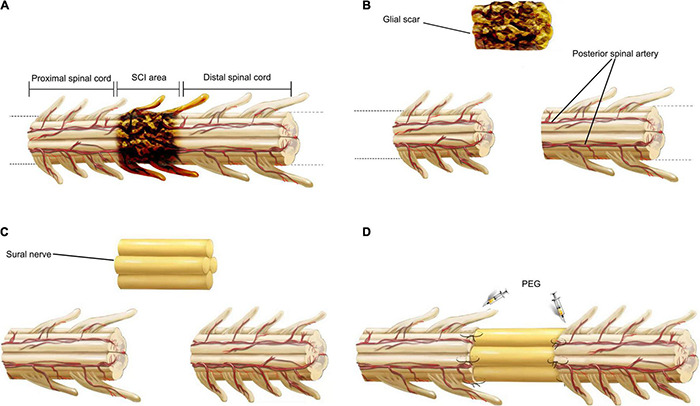
Key surgical steps of SNT. First, the area of spinal cord injury is removed to produce two acutely transected spinal cord stumps **(A,B)**. Then, based on the effective length of the defect between two fresh spinal stumps, a section of sural nerve harvested from the patient’s lower leg simultaneously, was divided into several bundles, maintaining the cranial and caudal orientation of all the bundles, and sutured side by side into one bundle **(C)**. Finally, the sural nerve is autotransplanted to bridge the gap between the distal and proximal spinal cords. The sites of transection were topically applied with PEG to complete the SCF, and the two sites of transection are then anastomosed with suturing under the microscope **(D)**.

The patient was kept in a prone position and under general anesthesia. The skin and muscles overlying the thoracic spinal column were incised. A laminectomy was performed at the SCI level with a cutting ultrasonic scalpel (BoneScalpel^®^, Misonix, United States) to expose the dura mater, which was then cut open to expose the spinal cord. Then, SNT was performed. The main surgical operations are shown in Figure 1. The SCI area (Table 1) was removed using an extremely sharp scalpel (MANI, Japan) to produce two acutely and sharply transected spinal cord stumps (the removed spinal cord tissue was examined with immunohistochemical staining). The skin and subcutaneous fat overlying the sural nerve were incised. The sural nerve was excised from the patient’s lower leg, then divided into several bundles (4–8), maintaining the cranial and caudal orientation of all the bundles, and sutured side by side into one larger diameter bundle using a microsurgery suture thread with needle (7-0, COVIDIEN, United States). The sural nerve bundle graft was transplanted to bridge the distal and proximal spinal cord stumps. The diameter of the sural nerve bundle graft was close to that of the spinal cord stumps. The two sites of spinal cord transection produced after excision of the diseased section of the spinal cord were bathed in 10 ml of PEG (100%, Sigma-Aldrich/Merck, Germany) and anastomosed by suturing the ends of the sural nerve bundle graft under the microscope with a microsurgery suture thread with needle (7-0, COVIDIEN, United States) to complete the SCF. The dura mater was sutured with an artificial dural patch (Guanhao Biotech, China). A 3-hole silicone drainage tube was placed into the muscular layer of the wound. The wound was closed layer by layer.

**TABLE 1:** Clinical characteristics of the patients.

Patient ID	Sex	Age	Months post SCI	SNL	Length of SCI tissue (cm)
SCF001	Female	47	28	T10	5
SCF002	Male	21	24	T12	7
SCF003	Male	38	51	T10	5
SCF004	Male	37	39	T12	7
SCF005	Male	46	15	T12	6
SCF006	Male	46	53	T10	6
SCF007	Male	33	26	T10	7
SCF008	Female	49	12	T6	7
SCF009	Male	38	48	T11	5
SCF010	Female	38	42	T10	8.5
SCF011	Male	44	21	T10	6
SCF012	Male	23	27	T3	4
Mean ± SD	–	38.33 ± 9.06	32.17 ± 14.01	–	6.13 ± 1.25

*SCF, spinal cord fusion; SCI, spinal cord injury; SNL, single neurologic level.*

### Safety Evaluation

After surgery, body temperature was measured three times a day. The site of the surgical incision was checked for any sign of infection once a day. Symptoms of cerebrospinal fluid leakage, such as headaches and dizziness, were closely observed. Potential adverse reactions to PEG, such as urticaria, dermatitis, and anaphylactic shock, were also monitored.

### Postoperative Rehabilitation

All patients received a series of rehabilitation programs involving specific exercises after the surgery. Two weeks after surgery and a professional assessment of the adequacy of each patient’s spinal stability, all patients began a series of instrument-assisted exercises. These exercises included use of an exoskeleton robot (AiWalker, Beijing Ai-Robotics Technology Co., Ltd., China) to assist walking (twice a day, 30 min each time), an intelligent auxiliary mobile robot (BangBang, Shanghai Bangbang Robot Co., Ltd., China) or paraplegic standing frame to keep the patient standing (twice a day, 40 min each time), and a rehabilitation bicycle to exercise the joints of the patient’s lower extremity passively (twice a day, 1 h each time). These devices enabled patients to stand and walk in the early postoperative period to the best of their ability, which helped to stimulate the cerebral cortex to readjust, control the peripheral nerves of the lower extremities, and coordinate the movements of the lower extremity.

### Immunohistochemistry

The removed spinal cord tissue was fixed with 4% paraformaldehyde for 24 h and then embedded in paraffin and horizontally cut into transverse slices of 7 μm thickness. After removing the paraffin, the slices were quenched with endogenous peroxidase activity in 3% methanol hydrogen peroxide for 0.5 h. The antigen^2^ was fixed in the citrate buffer (pH 6.0) in a microwave for 5 min. After being blocked in 5% goat serum/PBS with Triton for 30 min at 37°C, the slices were incubated with the primary antibodies (NF-200, 1:50, Cat#: ab19386 and MBP, 1:50, Cat#: ab 2404, both from Abcam, United States) overnight at 4°C, and then immersed with the biotinylated secondary antibodies (PV secondary antibody kit, Beijing Zhongshan Jinqiao Biotechnology Co., Ltd., China) for 0.5 h at room temperature. After incubating with peroxidase-conjugated streptavidin, the immune complexes were visualized by incubating slices in the DAB system. Finally, the slices were covered with coverslips. The spinal cord slices were imaged at 400x using an Olympus microscope (Olympus IX73, Tokyo, Japan). The axons of the spinal cord were stained by the NF-200 specific antibody. The myelin sheaths were stained by an antibody specific for myelin basic protein (MBP).

### Pain Assessment

The visual analog scale (VAS) is one of the most widely used methods to give valid and reliable assessments on experimental pain as well as acute and chronic pain in patients. The VAS consists of a 10 cm horizontal line on a card with the words “no pain” and “worst pain ever” placed at the left and right hand extremes of the line, respectively. The patients were instructed to mark the line at a point representing their pain intensity (Ekblom and Hansson, 1988).

### Neurological Assessment

Neurological evaluations of enrolled patients with paraplegia were performed according to the International Standard of Neurological Classification for Spinal Cord Injury (ISNCSCI)/American Spinal Cord Injury Association (ASIA) classification of SCI (Maynard et al., 1997). The ASIA impairment scale (Table 2) and ISNCSCI motor score, pin prick, and light touch scores were assessed.

**TABLE 2:** ASIA impairment scale (Maynard et al., 1997).

ASIA impairment scale	
**A**	Complete. No sensory or motor function is preserved in the sacral segments S4-S5
**B**	Incomplete. Sensory but not motor function is preserved below the neurological level and includes the sacral segments S4-S5.
**C**	Incomplete. Motor function is preserved below the neurological level, and more than half of key muscles below the neurological level have a muscle grade of less than 3.
**D**	Incomplete. Motor function is preserved below the neurological level, and at least half of key muscles below the neurological level have a muscle grade greater than or equal to 3.
**E**	Normal. Sensory and motor function is normal.

### Neurophysiological Assessment

To assess the conduction of the spinal cord action potentials, different electrophysiological variables, including motor evoked potentials (MEPs) and somatosensory evoked potentials (SSEPs), were examined before surgery and at 1, 3, and 6 months after surgery.

SSEPs were recorded and analyzed with the NIM-ECLIPSE^®^ System (Medtronic, United States). Cortical SSEPs were elicited by a 200 μs squarewave electrical pulse presented sequentially to the median and posterior tibial nerves. The stimulus frequencies were 3.96 Hz with an intensity 20 and 30 mA to the median and posterior tibial nerves, respectively. Cortical potentials were recorded from monopolar needle electrodes placed at Cz’ for posterior tibial nerve stimulation and at C3′ or C4′ for median nerve stimulation, and referenced to Fpz (Costa et al., 2007) (international 10–20 EEG system).

MEPs were recorded and analyzed in the QuickStim System (YINGCHI, China). MEPs were elicited by 60% transcranial magnetic stimulation using the magnetic stimulator (YINGCHI, China) of primary motor cortex and recorded in the contralateral abductor pollicis brevis in the upper extremities and tibialis anterior in the lower extremities.

### Neuroimaging Assessment

All patients were subjected to MRI and DTI (Jones and Leemans, 2011; Hendrix et al., 2015; D’Souza et al., 2017) using a 1.5 T MRI system (Ingenia 1.5, Philips, Amsterdam, The Netherlands) in the supine position. Sagittal, T2-weighted, fast spin-echo (TR = 2,500 ms; TE = 110 ms; slice thickness = 4 mm; slice gap = 0.4; NSA = 2) and axial single-shot echo-planar DTI (TR = 7,193 ms; TE = 81 ms; voxel size = 2.34 mm × 2.46 mm; slice thickness = 5 mm; slice gap = 0.06; FOV = 280 mm x 280 mm; NSA = 4; diffusion direction number = 15) sequences were acquired preoperatively and 1, 3, and 6 months postoperatively in all patients. DTI original images were processed to produce color DTI maps.

After the image collections, the data were analyzed quantitatively and qualitatively. For qualitative analysis, a region of interest (ROI) was drawn in the spinal cord. This became the seed point for automatically generating the entire white matter tract in the spinal cord segment included in the FOV through the inbuilt software. To evaluate the quantitative index, an ROI (with a size of 3 mm) was drawn into the white matter column of the spinal cord. Special attention was paid to avoid partial volume averaging with underlying gray matter and overlying cerebrospinal fluid. In the inbuilt software, automatic calculations of DTI indices, including fractional anisotropy (FA) and the apparent diffusion coefficient (ADC), were performed within the ROI (D’Souza et al., 2017).

### Statistical Analysis

All data were analyzed using SPSS Statistics software (SPSS, IBM, New York, United States). Data are presented as the mean ± SD. Statistical analysis was performed with a paired sample *t*-test. Statistical significance was set at *p* < 0.05.

## Results

### Patient Enrollment and Characteristics

**FIGURE 2:**
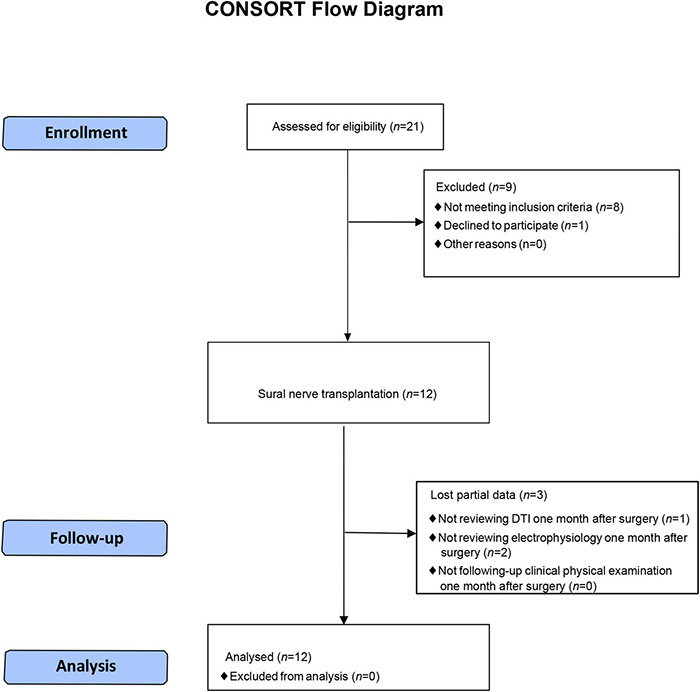
CONSORT flow diagram.

A total of 12 paraplegic patients (nine male and three female; mean age, 38.33 ± 9.06 years; mean duration since injury, 32.17 ± 14.01 months) were enrolled in the study (Table 1 and Figure 2). The entire operation of SNT took approximately 4 h to complete. After the surgery, all patients recovered during the perioperative period safely without complications such as high fever, wound infection, and cerebrospinal fluid leakage. None of the patients had clinically important adverse reactions to PEG up to 6 months after the surgery.

### Immunohistochemistry

**FIGURE 3:**
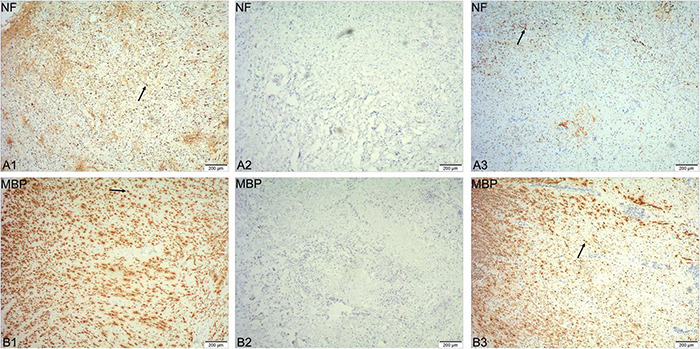
Immunohistochemical staining of spinal cord tissue removed during the surgery. NF-200 positive axons and MBP-positive myelin sheaths can be observed at the distal and proximal ends of the tissue (arrows in **A1,B1,A3,B3**), but not in the center of the tissue **(A2,B2)**.

The spinal cord tissue removed during the surgery was stained with immunohistochemical staining. The slices were imaged at 40x using an Olympus microscope (Olympus IX73, Tokyo, Japan). Immunohistochemistry showed that NF-200-positive axons and MBP-positive myelin sheaths could be observed at the distal and proximal ends of the tissue (Figures 3A1,A3,B1,B3), but no such immunopositivity was ever evident in the center of the tissue (Figures 3A2,B2). This observation indicated that the neural continuity of the spinal cord was completely interrupted, and that the entire area of the SCI was completely removed by the surgery.

### Pain Assessment

**FIGURE 4:**
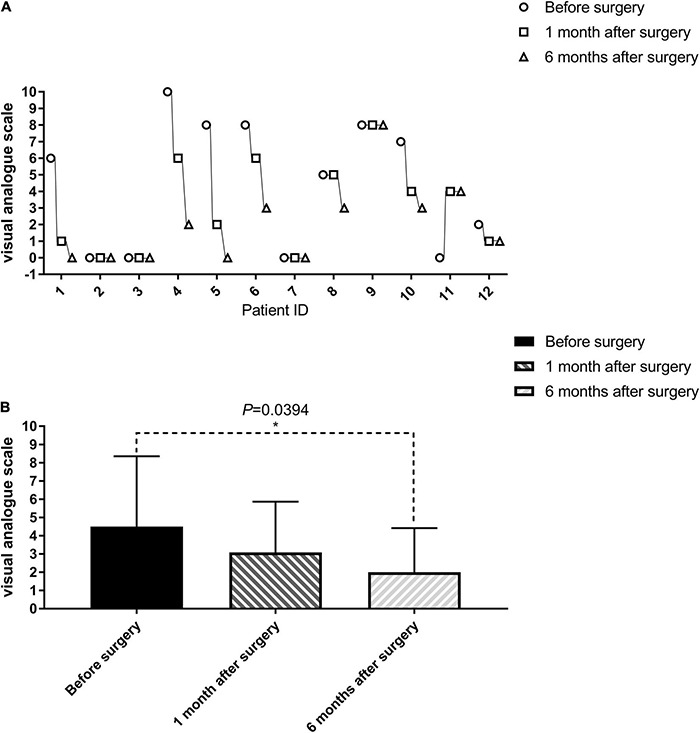
Evaluation of cord central pain in patients with paraplegia before, 1 and 6 months after the surgery (visual analog scale, VAS). The VAS of one patient (SCF011) who had no spinal central pain before the surgery developed after the SNT. The VAS of seven patients (SCF001, SCF004, SCF005, SCF006, SCF008, SCF010, SCF012) after the surgery gradually decreased compared with that before the surgery. The remaining patients were unchanged (1–12 on the horizontal axis represents SCF001-SCF012, see in **(A)**. The difference between VAS before and 6 months after the surgery was statistically significant by a paired sample *t*-test, *p* < 0.05 **(B)**.

Pain is one of the main and most intractable complications of paraplegia patients and seriously affects the patient’s quality of life (Westgren and Levi, 1998). The most common type of pain experienced was cord central pain, which is a chronic neuropathic pain disorder that develops as a direct consequence of a lesion within the central nervous system (Wasner, 2010). The incidence of cord central pain after SCI has been estimated to range from 20 to 40% (Canavero and Bonicalzi, 2018). Eight of the 11 patients with paraplegia in this clinical trial had some element of cord central pain symptoms; indeed 4 of these 8 patients graded the severity of their symptoms as 8 or more out of 10 on the VAS scale (Table 3). Surgery did not cause or worsen cord central pain symptoms in four patients. Patient SCF011, who had no cord central pain symptoms before the surgery, developed postoperative pain symptoms graded as a 4 on the VAS scale. However, seven paraplegic patients with cord central pain had quite marked relief of pain symptoms after the surgery (Table 3 and Figure 4A). The mean VAS of all patients at 1 and 6 months after the surgery gradually decreased compared with that before the surgery (3.09 at 1 month after the surgery vs. 2.00 at 6 months after the surgery vs. 4.50 before the surgery, Table 3 and Figure 4). Although there was no statistically significant difference in VAS before and 1 month after the surgery (paired sample *t*-test; *p* = 0.1014), the difference between VAS before and 6 months after the surgery was statistically significant (paired sample *t*-test; *p* < 0.05; Figure 4B).

**TABLE 3:** Visual analog scale for cord central pain in paraplegic patients.

Patient ID	VAS		
	Before surgery	One month after surgery	Six months after surgery
SCF001	6	1	0
SCF002	0	0	0
SCF003	0	0	0
SCF004	10	6	2
SCF005	8	2	0
SCF006	8	6	3
SCF007	0	0	0
SCF008	5	5	3
SCF009	8	8	8
SCF010	7	4	3
SCF011	0	4	4
SCF012	2	1	1
Mean ± SD	4.50 ± 3.85	3.09 ± 2.78	2.00 ± 2.41

*SCF, spinal cord fusion; VAS, Visual analog scale.*

### Neurological Assessment

The patients were evaluated for autonomic function after surgery. Seven patients recovered sweating below the single neurologic level. Two patients had improved bladder and/or bowel function. The stool characteristics of patients SCF001 and SCF005 were improved from dry and hard to wet and soft, and the symptoms of urinary incontinence in patient SCF004 were improved (Table 4). In addition, before the surgery, all patients had complete loss of sensory and motor functions below the single neurologic level. After SNT, motor functions were restored to varying extents in four patients. Patient SCF001 was able to autonomously flex and extend the ankle and toe joints of both lower extremities 1 month after the surgery. Patient SCF005 was able to autonomously flex his hip joints and extend his knee joints 2 months after the surgery. Because patient SCF005 is a patient with lower thoracic SCI, he could move slowly along the parallel bars through the support of the upper limbs and the rotation of the body before the operation, but could only move for a few minutes. After the operation, because his iliopsoas and quadriceps muscles recovered a certain amount of muscle strength, he could walk quickly along the parallel bars, and his gait was closer to normal. He could also walk for 1 h each time (Supplementary Video 1). Patient SCF010 was able to autonomously flex her hip joints 1 month after the surgery. Patient SCF0012 was able to autonomously flex the ankle joints of both lower extremities and extend toe joints of both lower extremities 3 months after the surgery, but not to a great of useful extent.

**TABLE 4:** The recovery condition of autonomic nerve function in paraplegic patients at 6 months postoperatively.

Patient ID	The recovery condition of autonomic nerve function
SCF001	Restored sweating function below the single neurologic level; bowel function improved
SCF002	–
SCF003	–
SCF004	Restored sweating function below the single neurologic level
SCF005	Restored sweating function below the single neurologic level; bladder and bowel function improved
SCF006	Restored sweating function below the single neurologic level
SCF007	–
SCF008	Restored sweating function below the single neurologic level
SCF009	–
SCF010	Restored sweating function below the single neurologic level
SCF011	–
SCF012	Restored sweating function below the single neurologic level

*SCF, spinal cord fusion.*

In addition, the sensory function also showed improvements in three patients. Patients SCF004, SCF005, and SCF010 had some recovery of superficial skin sensations below the single neurologic level (Figure 4). At 6 months after the surgery, when the surgeon moved the joints of the lower limbs of patients SCF004, SCF005 and SCF012, the patients could accurately identify which lower limb joint was moving, indicating the restoration of deep sensation below the single neurologic level.

**FIGURE 5:**
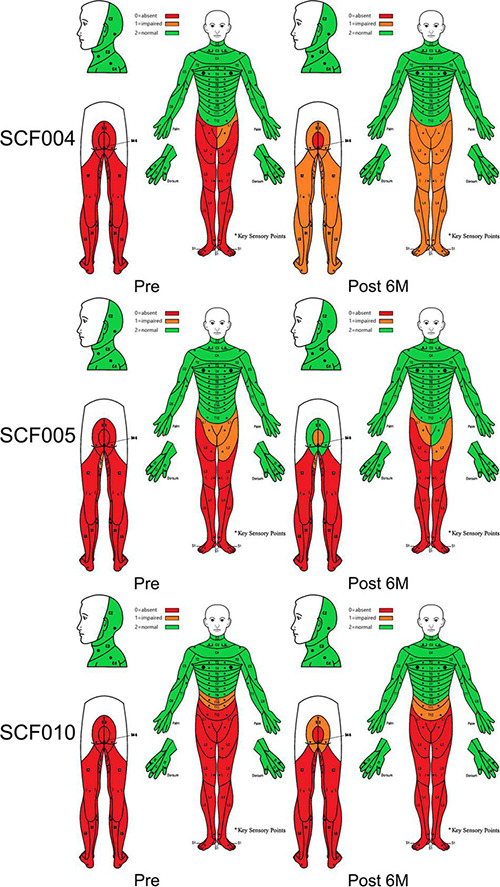
Dermatomal sensory scores according to the International Standards for Neurological Classification of Spinal Cord Injury before and at 6 months after the surgery.

According to the ISNCSCI/ASIA classification of SCI, all 12 patients were classified as ASIA Impairment Scale grade A before the surgery (the most severe). The ISCNSCI motor scores of all patients were 50 indicating lack of motor activity (upper: 50, lower: 0, Table 5). At 6 months post-surgery, the ISCNSCI lower extremity motor score of patient SCF001 was improved from 0 to 16, and the ASIA grade was improved from A to C. In patient SCF004, the ISCNSCI Light Touch scores and Pinprick scores were both improved from 81 to 98, and the ASIA grade increased from A to B. For patient SCF005, the ISCNSCI lower extremity motor score was restored from 0 to 8, the ISCNSCI Light Touch scores and Pinprick scores both were improved from 79 to 84, and the ASIA grade was improved from A to B. In patient SCF010, the ISCNSCI lower extremity motor score was improved from 0 to 6, the ISCNSCI Light Touch and Pinprick scores both improved from 68 to 72, and the ASIA grade improved from A to B. The ISCNSCI lower extremity motor score of patient SCF012 improved from 0 to 4, but the ASIA grade remained as A (Table 5 and Figure 5).

**TABLE 5:** Sensory and motor function assessments according to the International Standards for Neurological Classification of Spinal Cord Injury.

Patient ID	Before surgery						6 months after surgery					
	SNL	ISCNSCI UEMS	ISCNSCI LEMS	ISCNSCI light touch scores	ISCNSCI pinprick scores	ASIA grade	SNL	ISCNSCI UEMS	ISCNSCI LEMS	ISCNSCI light touch scores	ISCNSCI pinprick scores	ASIA grade
SCF001	T10	50	0	68	68	A	T10	50	16	68	68	C
SCF002	T12	50	0	76	76	A	T12	50	0	76	76	A
SCF003	T10	50	0	68	68	A	T10	50	0	68	68	A
SCF004	T12	50	0	81	81	A	T12	50	0	98	98	B
SCF005	T12	50	0	79	79	A	T12	50	8	84	84	B
SCF006	T9	50	0	66	66	A	T8	50	0	62	62	A
SCF007	T10	50	0	72	72	A	T8	50	0	66	66	A
SCF008	T6	50	0	56	56	A	T5	50	0	54	54	A
SCF009	T10	50	0	74	76	A	T10	50	0	74	76	A
SCF010	T9	50	0	68	68	A	T10	50	6	72	72	B
SCF011	T9	50	0	72	72	A	T9	50	0	72	72	A
SCF012	T3	50	0	51	51	A	T3	50	4	51	51	A

*SCF, spinal cord fusion; SNL, single neurologic level; ISCNSCI, International Standard of Neurological Classification for Spinal Cord Injury; UEMS, motor score of the upper extremities; LEMS, motor score of the lower extremities; ASIA, American Spinal Injury Association.*

### Neurophysiological Assessment

**FIGURE 6:**
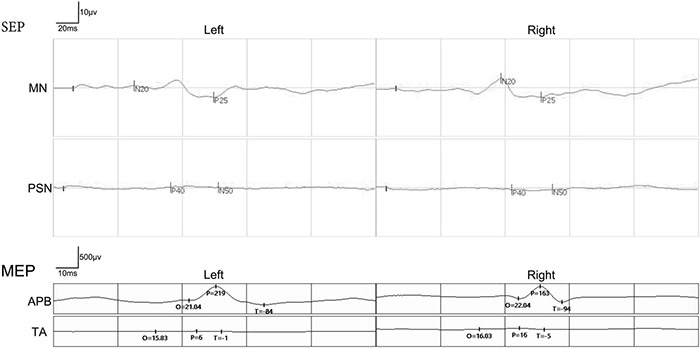
A representative picture of somatosensory evoked potentials (SSEPs) and motor evoked potentials (MEPs) before the surgery in the paraplegic patients. The SSEPs of the bilateral median nerve (MN) were normal. The SSEPs of bilateral posterior tibial nerve (PTA) were missing. MEPs recorded at abductor pollicis brevis (APB) in the bilateral upper extremities were normal. The MEPs recorded at both tibialis anterior (TA) in the bilateral lower extremities were missing.

**FIGURE 7:**
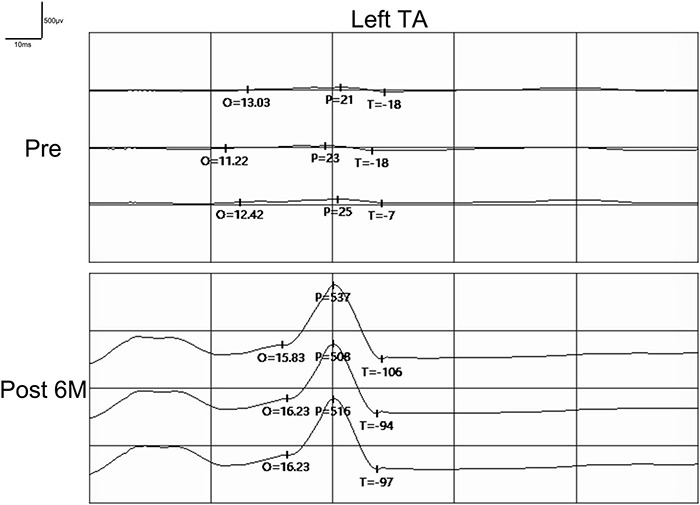
Pre- and postoperative MEPs recorded at tibialis anterior (TA) by transcranial magnetic stimulation in patient SCF010. MEPs recorded at left tibialis anterior showed signs of recovery postoperatively. Pre, Preoperatively; Post, Postoperatively; O, Latency; P, Amplitude.

Preoperatively, the SSEPs of the bilateral median nerve were normal in these 12 patients, while the SSEPs of bilateral posterior tibial nerve were completely absent as expected. In addition, the MEPs recorded at abductor pollicis brevis muscles in the bilateral upper extremities were normal, and the MEPs recorded at tibialis anterior muscles in the bilateral lower extremities were completely absent as expected (Figure 6). The preoperative SSEPs and MEPs of patient SCF010 were not different from those of the other 11 patients, but at 6 months post-surgery, the MEPs of this patient recorded at the left tibialis anterior indicated clinically relevant recovery (the right MEPs did not recover) (Figure 7). The remaining 11 patients showed no recovery of SSEPs and MEPs on the neurophysiological assessment postoperatively.

### Neuroimaging Assessment

**FIGURE 8:**
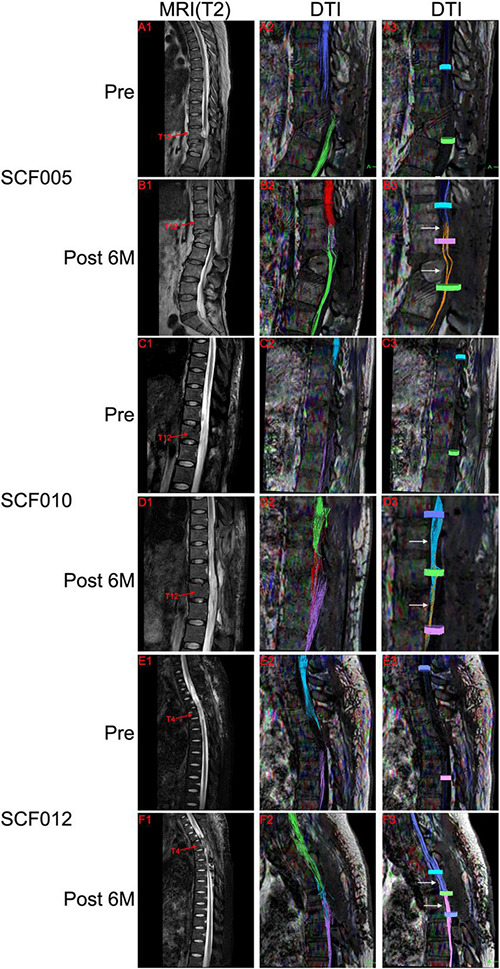
Representative neuroimages. Preoperative T2-weighted MRI scans showed spinal cord injury (**A1,C1,E1**), and DTI showed complete disruption of the spinal cord fibers (**A2,A3,C2,C3,E2,E3**). Postoperative T2-weighted MRI scans and DTI showed the autotransplanted sural nerve in the original spinal cord injury area (**B1,B2,D1,D2,F1,F2**). DTI showed restoration of neural connection at the two sites of transection (white arrows in **B3,D3,F3**). The colors of the fibers were automatically generated by the DTI system of MRI, with no practical significance. Pre, Preoperatively; Post 6M, 6 months postoperatively.

Preoperatively, the T2-weighted MRI scans of all patients showed substantial abnormal signal intensity in the area of SCI. In addition, DTI showed no neural continuity between the proximal and distal spinal cords in these patients. MRI and DTI were repeated on all patients after the surgery. T2-weighted MRI scans of all patients showed the autotransplanted sural nerve tissue at the original SCI site. In addition, the postoperative DTI of patients SCF001, SCF002, SCF005, SCF006, SCF010, SCF011, and SCF012 showed neural connections at the two sites of transection (Figure 8 and Supplementary Figure 1). In the remaining five patients, two patients showed neural connection at the site of the proximal transection but not at the distal transection in DTI. One patient showed neural connection at the site of the distal transection but not at the proximal transection in DTI. The postoperative DTI of the remaining two patients showed no neural connection at either site of transection (Supplementary Figure 2).

**FIGURE 9:**
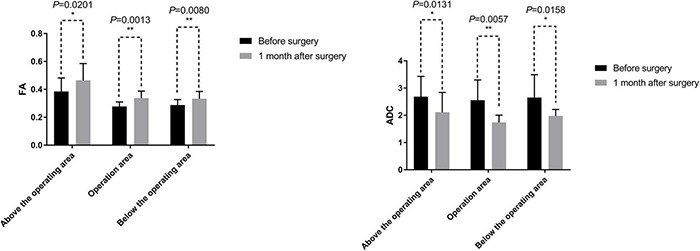
DTI related measurements include fractional anisotropy (FA) and apparent diffusion coefficient (ADC). The mean values of FA at 1 month after the surgery were greater than before the surgery. The difference of FA values before and 1 month after the surgery was statistically significant by a paired sample *t*-test (*p* < 0.05). The mean values of ADC at 1 month after the surgery were less than that before the surgery. The difference of ADC values before and 1 month after the surgery was statistically significant by a paired sample *t*-test (*p* < 0.05). *, 0.01 < *p* < 0.05; **, *p* < 0.01.

In addition, the related variables of DTI in all paraplegic patients, FA and ADC, also showed some improvements. At 1 month after the surgery, the average FA values were increased compared with those before the surgery, while the average ADC values decreased compared with those before the surgery (Table 6 and Figure 9). The difference in the FA values in the operation area before and 1 month after the surgery was statistically significant (paired sample *t*-test; *p* < 0.05). The difference of ADC values above the operation area before and 1 month after the surgery was also statistically significant (paired sample *t*-test; *p* < 0.05) (Table 6 and Figure 9).

**TABLE 6:** Statistical analysis of DTI data parameters.

DTI Parameter		Above the operating area	Operation area	Below the operating area
FA (mean ± SD)	Before surgery	0.39 ± 0.10	0.28 ± 0.03	0.29 ± 0.04
	One month after surgery	0.47 ± 0.12	0.34 ± 0.05	0.33 ± 0.05
	*p*-value	0.0201*	0.0013**	0.0080**
ADC (mean ± SD)				
	Before surgery	2.69 ± 0.74	2.55 ± 0.74	2.65 ± 0.84
	One month after surgery	2.11 ± 0.72	1.74 ± 0.27	1.98 ± 0.24
	*p*-value	0.0131*	0.0057**	0.0239*

*p < 0.05.

*DTI, Diffusion Tensor Imaging; FA, Fractional anisotropy; ADC, Apparent diffusion coefficient.*

## Discussion

According to the rationale and principle of the GEMINI SCF protocol, an acutely and sharply transected thoracic spinal cord (no gap between two spinal cord stumps) can be fused in the presence of topical PEG. Our team has demonstrated evidence to support this rationale and principle in several different small and large animal models (Ye et al., 2016; Ren et al., 2017, Liu et al., 2018; Ren et al., 2019; Ren and Canavero, 2020). However, in clinical patients with SCI, there are glial scars between the two spinal cord stumps. After SCI, astrocytes, the most abundant glial cells in the central nervous system, are transformed into reactive astrocytes due to changes in the local microenvironment. The glial scar (mainly astrocytic) evolves from the dynamic process of reactive gliosis and builds a physical barrier to axonal growth (Yiu and He, 2006; Bradbury and Burnside, 2019). Another important feature of glial scars is the increased expression of components of the extracellular matrix (ECM), which are secreted by reactive astrocytes. Among the ECM components, chondroitin sulfate proteoglycans (CSPGs) have been shown to inhibit axon regeneration and plasticity (Yuan and He, 2013). Currently, some studies have proposed surgical resection to remove the glial scar (Goldsmith et al., 2005; Tabakow et al., 2014; Xiao et al., 2016). However, surgical removal of the scar also results in a gap of varying lengths between the two spinal stumps. Therefore, our team proposed a graft of vascular pedicle hemisected spinal cord transplantation (vSCT), in which half of the spinal cord tissue with one side of the posterior spinal artery is cut from the distal or proximal spinal cord tissue site and transplanted to the gap to bridge the distal and proximal spinal cord stumps. The other side of the posterior spinal artery is maintained as a vascular pedicle to supply blood flow to the transplanted spinal cord graft. With this approach, two sites of spinal cord transection were produced after transplantation. However, when the level of SCI is in the lower thoracic area (the SCI area is close to the cone), or when the level of SCI is not in the lower thoracic area but the distal spinal cord in the area of the SCI is markedly atrophied, cutting half of the spinal cord tissue from the distal spinal cord tissue site and rotating a vascularized graft to the gap (vSCT) is not feasible. Additionally, cutting half of the spinal cord tissue from the proximal spinal cord tissue site led to the ascent of the single neurologic level. Therefore, in our current work, we chose another clinical translational model (SNT) to treat these types of patients. The sural nerve was excised from the patient’s lower leg and then divided into several bundles, maintaining the cranial and caudal orientation of all the bundles, and sutured side by side into one bundle. The sural nerve was transplanted to bridge the distal and proximal spinal cord stumps. Furthermore, axonal disintegration would be expected to start after about 10–20 min after the transection (Seif et al., 2007) and thus PEG was applied immediately on the transection sites to have its sealant/fusogen effects topically (Canavero et al., 2016).

One of the key conditions for the success of the GEMINI SCF protocol is that the fused spinal cord stumps must be acutely and sharply transected, bathed in the PEG fusogen, and be well apposed without gaps. During the operation, we used an extremely sharp scalpel (MANI, Japan) to excise the glial scar of the spinal cord as atraumatically as possible. The extremely sharp scalpel was also used to cut and design the sural nerve to minimize damage. The SCI caused by this extremely sharp scalpel is very different from the SCI caused by external trauma. Studies have shown that the typical force generated by rapid transection of the spinal cord with an extremely sharp scalpel is less than 10 N, while the force generated by clinical SCI is about 26,000 N, a difference of 2,600 times (Sledge et al., 2013; Canavero et al., 2016). In addition, we obtained the sural nerve with the length slightly longer than the gap between the two cord stumps to ensure that the sural nerve and spinal cord were well apposed. This experimental approach provides favorable conditions for PEG to promote the fusion of the axon stump in the sural nerve and spinal cord.

DTI has been reported as a novel technique for imaging white matter and peripheral nerve fibers (Cauley and Filippi, 2013; Simon et al., 2016; D’Souza et al., 2017; Palacios et al., 2020). In this clinical trial, we demonstrated that the autotransplanted sural nerve bundles could be imaged and their anatomic continuity traced by DTI. Consistent with the results of our previous animal experiments (Ye et al., 2016; Ren et al., 2017, Liu et al., 2018; Ren et al., 2019; Ren and Canavero, 2020), the postoperative DTI of patients SCF001, SCF002, SCF005, SCF006, SCF010, SCF011, and SCF012 showed the establishment of anatomic neural connections at the two sites of transection. These findings indicated that SNT successfully allowed membrane fusion of the axonal stumps of the spinal cord white matter and the sural nerve. In the remaining five patients, two patients showed neural connection at the site of the proximal transection but not at the distal transection. One patient had neural connection at the site of the distal transection but not at the proximal transection. The postoperative DTI of the remaining two patients showed no neural connection at either site of transection. In some patients, we needed to use absorbable rivets to fix the artificial dural patch in place to repair the dura during the operation; these rivets prevented the ability to use postoperative DTI imaging, so that the neural connection at the sites of transection could not be observed. In addition and importantly, DTI showed that the autotransplanted sural nerve without any feeding vascular blood supply survived at the operation area. Unfortunately, DTI cannot image gray matter and thus we could not show direct neural continuity of the gray matter across the site of transection. However, the return of some motor activity distal to the sites of transection certainly suggests that there was gray matter reinnervation.

In addition to imaging and tracing neural continuity of the spinal cord and the sural nerve connections, the DTI can also be used to quantitatively evaluate the spinal cord by calculating the DTI anisotropy indices (datametrics). The indices of datametrics can quantify any observed changes, with the two most common being FA and ADC. FA and ADC were used to infer tissue characteristics and tissue physiology of the spinal cord (Basser and Pierpaoli, 2011). FA reflects the anisotropy of diffusion. FA is considered a marker of white matter integrity, and any abnormality in the FA indicates injury or degeneration of the white matter of the spinal cord (Sasiadek et al., 2012). ADC reflects the magnitude of diffusion. A low ADC value indicates that the structure of the white matter (such as nerve fibers) is organized, whereas a high ADC value indicates that the neural structure is disorganized (Sledge et al., 2013). In the present trial, compared with the preoperative values, the postoperative FA values increased, but the ADC values decreased. These observations suggested that conditions of the SNT treatment had a certain neuroprotective effect, reduced the degree of nerve fiber injury and degeneration, and made the nerve fiber structure of the sural nerve better and more organized.

During the neurological assessments, some patients showed signs of functional neurological recovery after SNT, including autonomic, motor, and sensory function. For example, seven patients recovered sweating below the single neurologic level. Two patients had improved bladder and bowel function. Patients SCF001, SCF004, SCF005, SCF010, and SCF012 regained some somatosensory and/or motor function below the single neurologic level. The MEPs of patient SCF010 recorded at the left tibialis anterior recovered. As mentioned above, DTI can only trace and image white matter and peripheral nerve fibers, but not gray matter. Therefore, we hypothesize that, after SCF, in addition to restoration of neural continuity in the spinal white matter, there might be additional restoration of neural continuity in terms pathways of neural signaling in the gray matter in the spinal cord to support the restoration of motor and sensory functions in paraplegics.

As mentioned in the GEMINI SCF protocol, there are two sets of fiber tracts from the brain to the spinal cord that regulate voluntary motor and sensory function of the extremities (Canavero et al., 2016; Ren et al., 2019). One is the cortico-trunco-reticulo-propriospinal (CTRPS) pathway from the brain to the spinal cord, which connects neurons through short range fibers from the cerebral cortex to the spinal cord. Studies confirmed that this intraspinal network of propriospinal neurons plays a critical role in motor reflexes, voluntary movement, and sensory processing, as well as in the functional recovery after SCI (Flynn et al., 2011). As the species evolved, however, a much faster transmission system, the pyramidal tract developed, consisting of long, fast-transmitting neurons that connect nerve cells in the cortex of the brain to those in the spinal cord, allowing for the rapid transmission of the signals. After the spinal cord is severed, the damaged long nerve fibers can only recover if ever, very slowly, because regeneration of nerves must take place from the brain to the spinal cord. In contrast, propriospinal neurons based on the gray matter have a high regeneration response (Flynn et al., 2011). In incomplete thoracic SCI, the corticospinal tract fibers in the pyramidal tract sprout into the gray matter of the cervical spinal cord and connect with the descending propriospinal neurons. The long propriospinal neurons increase their terminal arborizations onto the lumbar motor neurons to form synapses, thus creating a new neural circuit (detour) (Bareyre et al., 2004; Filli et al., 2014). However, in complete thoracic SCI, combined with the GEMINI SCF protocol, we believe that in addition to the above propriospinal neural circuit (detour), the SCF also plays a vital role. We propose that, after SNT, the neuroprotective fusogen PEG allows fusion of the distal and proximal spinal cord white matter stumps and the sural nerve to establish an early direct neural connection at the two sites of transection. This has been demonstrated by DTI in some patients in this clinical trial (Figure 8). However, DTI did not show neural connection at the sites of transection (proximal or/and distal) in some patients. Considering that DTI could not image the gray matter, we proposed that after SNT, these propriospinal neurons in the gray matter of the spinal cord stumps undamaged by the extra-sharp blade could regrow (sprout) their fibers immediately and make contacts with the autotransplanted sural nerve to re-establish the neural continuity. In addition, PEG also could fuse the axon stumps of the propriospinal neurons and sural nerve to re-establish neural continuity. This may be an explanation for the DTIs of patients SCF001, SCF004, SCF005, SCF010, and SCF012. These patients had no restoration of overall continuity in the white matter, but still had partial sensory and/or motor function recovery below the single neurologic level. In our previous animal study, a beagle regained motor function of the lower limbs and restored the cBBB score (from 0 to 11), even though DTI showed no white matter neural continuity after surgery (Unpublished data).

In this clinical trial, seven of the 12 SNT-treated paraplegic patients did not yet show clinically relevant recovery of sensory and/or motor functions. A longer observation is important, because restoration of cord neural continuity is a prerequisite for recovery of lost sensory and motor functions below the single neurologic level. According to the postoperative DTI and electrophysiological results of our patients, combined with the CTRPS pathway theory mentioned in the GEMINI SCF protocol, we cannot rule out the possibility that the patients might restore the cord neural continuity through the CTRPS pathway. The paraplegic patients in the clinical trial all had chronic spinal cord injuries, with the duration ranging from 1 to 4 years, and had varying degrees of atrophy of the spinal cord and muscles. Therefore, if the experimental conditions of SNT did allow the fusion of the axons of the two sites of spinal transection with those of the sural nerve bundle, thereby restoring neural continuity of the spinal cord, the patients’ sensory and motor functions below the single neurologic level might require a longer recovery time. In addition, the limited number of sensory and motor fibers that were fused by SNT were likely mismatched, and thus might require a longer time for adaptation and reorganization of these pathways to convey the signal from the brain for motor function and from the periphery for sensory function. Indeed, recovery from any anatomic disruption of the spinal cord utilizes the entire central nervous system, namely, the cord, brainstem, and brain, in which a massive degree of reorganization (large-scale “rewiring”) occurs (Isa and Nishimura, 2014). In 2005, Goldsmith et al. (2005) reported a 24-year-old woman who had a high-speed skiing accident that caused a complete traumatic anatomical transection of her spinal cord at the T6-T7 level. During the surgery 39 months after the SCI, the extensive glial scar at the injury site (approximately 4 cm) was completely removed. Subsequently, a solution/cytoskeleton of semi-liquid collagen was placed in the space between the proximal and distal spinal cord stumps, and an omental pedicle was placed directly on the collagen structure. At 6–8 months after the surgery, the patient experienced gluteal muscle activity, and motor activity extended into her legs, allowing her to walk with a walker and soft knee braces 4 years after the surgery. In 2014, Tabakow et al. (2014) reported a 38-year-old male with a traumatic spinal cord transection at T9 and clinical symptoms of complete SCI (ASIA A). At 21 months after the SCI, the glial scar was removed and the cultured olfactory ensheathing cells and olfactory nerve fibroblasts were seeded onto the spinal cord stump above and below the injury site. Four autologous sural nerves were used to bridge the 10 mm gap between the spinal cord stumps. Five months after the surgery, the patient began to show signs of recovery in sensory and motor functions of the lower extremities. The patient was able to perform lower extremity adduction, hip flexion, and knee extension about 1 year after the operation. In our current clinical trial, the average length of SCI in the paraplegic patients was 6.13 cm, which was much longer than the lengths of SCI in the above two patients. Although some patients did not show sensory and/or motor function recovery 6 months after the surgery, a longer follow-up time might be necessary to observe the functional recovery in these patients.

Another consideration is cord central pain, one of the most serious complications of paraplegia. In this clinical trial, we found an interesting but not wholly expected effect of SNT in relieving the cord central pain. Eight of the 12 paraplegics had symptoms of cord central pain preoperatively. After SNT treatment, the adherent scar tissue was completely removed and a rhizotomy of the affected nerve roots at the SCI site was performed. Seven out of these eight patients had their pain relieved to varying degrees, especially for patients SCF004 and SCF005. This success suggested that SNT treatment not only partially restored lost sensory and motor functions below the single neurologic level of paraplegic patients but also decreased the central nervous pain rather dramatically in several patients. One patient (SCF011), who had no cord central pain symptoms did, however, develop postoperative pain symptoms, which might be due to the damage to the patient’s proximal normal spinal cord tissue and nerve roots during the surgical removal of the scar.

In summary, this clinical trial demonstrated the clinical feasibility and safety of SNT in 12 paraplegic patients. First, we found that removal of scar tissue and a rhizotomy of the affected nerve roots at the SCI site during the SNT may be responsible for relief of cord central pain in some patients. The permanent loss of sensory and motor functions below the single neurologic level in patients with paraplegia after SCI was not restored because cord neural continuity was interrupted with the SCI. Further formation of scar tissue also completely blocks the pathway of nerve regeneration. In our study, in some patients, the sural nerve axons in the autotransplanted sural nerve bundles could be fused and connected neurophysiologically with the axons of distal and proximal spinal cord stumps, respectively. Although the DTI results showed that the white matter of the spinal cord did not restore the overall continuity, some patients had recovered partial sensory and motor function as well as autonomic nerve function. Because of the inability of DTI to image gray matter and considering the GEMINI SCF protocol, we believe that SNT restored the neural continuity of the CTRPS, thereby recovering certain motor and sensory functions. Long-term follow-up is necessary to understand whether some degree of functional adaptation will occur in the central nervous system. Finally, in the next clinical trial, we will optimize fusogen (PEG) and apply electrical stimulation, in an attempt to improve the surgical outcomes. We will also optimize surgical instruments to reduce surgical damage, thereby minimizing postoperative scar formation. In addition, allograft transplantation of spinal cord allografts as opposed to sural nerve autograft bundles might be another option during SCF to treat paraplegic patients, but further research is needed (Canavero et al., 2021).

## Data Availability Statement

The original contributions presented in the study are included in the article/Supplementary Material, further inquiries can be directed to the corresponding author/s.

## Ethics Statement

The studies involving human participants were reviewed and approved by the Medical Ethics Committee of Ruikang Hospital, affiliated with the Guangxi University of Traditional Chinese Medicine. The patients/participants provided their written informed consent to participate in this study. Written informed consent was obtained from the individual(s) for the publication of any potentially identifiable images or data included in this article.

## Author Contributions

XR contributed to conception and design of the study. WZ, LH, and RL organized the data acquisition. WZ and SR performed the statistical analysis. WZ wrote the first draft of the manuscript. XR, JQ, JM, YC, RL, and SR wrote sections of the manuscript. All authors contributed to manuscript revision, read, and approved the submitted version.

## Conflict of Interest

The authors declare that the research was conducted in the absence of any commercial or financial relationships that could be construed as a potential conflict of interest.

## Publisher’s Note

All claims expressed in this article are solely those of the authors and do not necessarily represent those of their affiliated organizations, or those of the publisher, the editors and the reviewers. Any product that may be evaluated in this article, or claim that may be made by its manufacturer, is not guaranteed or endorsed by the publisher.

## Footnotes

1
http://www.chictr.org.cn/index.aspx

2
https://www.abcam.com/protocols/ihc-fixation-protocol

## Supplementary Material

Supplementary Video 1 After the surgery, patient SCF005 walked while holding the parallel bars for exercise.
