# Supplementary material for: Partial Restoration of Spinal Cord Neural Continuity via Sural Nerve Transplantation Using a Technique of Spinal Cord Fusion
Source: Front Neurosci. 2022 Feb 14;16:808983. doi: 10.3389/fnins.2022.808983 (PMC8882688; doi:10.3389/fnins.2022.808983)
Supplement: Supplementary file 2 [file Image_1.pdf]

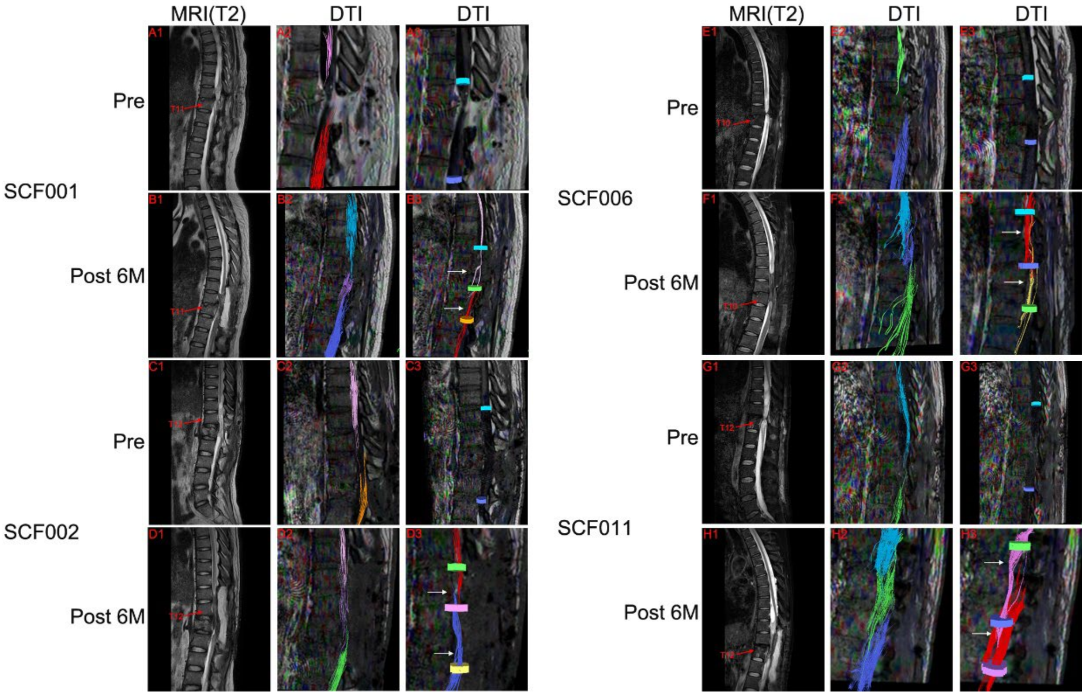

Fig.S1 Neuroimages of patients SCF001, SCF002, SCF006, and SCF011. Preoperative T2-weighted MRI scans show spinal cord injury (A1, C1, E1, G1), and DTI shows complete disruption of the spinal cord fibers (A2, A3, C2, C3, E2, E3, G2, G3). Postoperative T2-weighted MRI scans show the autotransplanted sural nerve in the original spinal cord injury area (B1, D1, F1, H1). DTI shows restoration of neural connection at the two sites of transection (white arrows in B3, D3, F3, H3). The colors of the fibers were automatically generated by the DTI system of MRI, with no practical significance. Pre: Preoperatively; Post 6M: 6 months postoperatively.

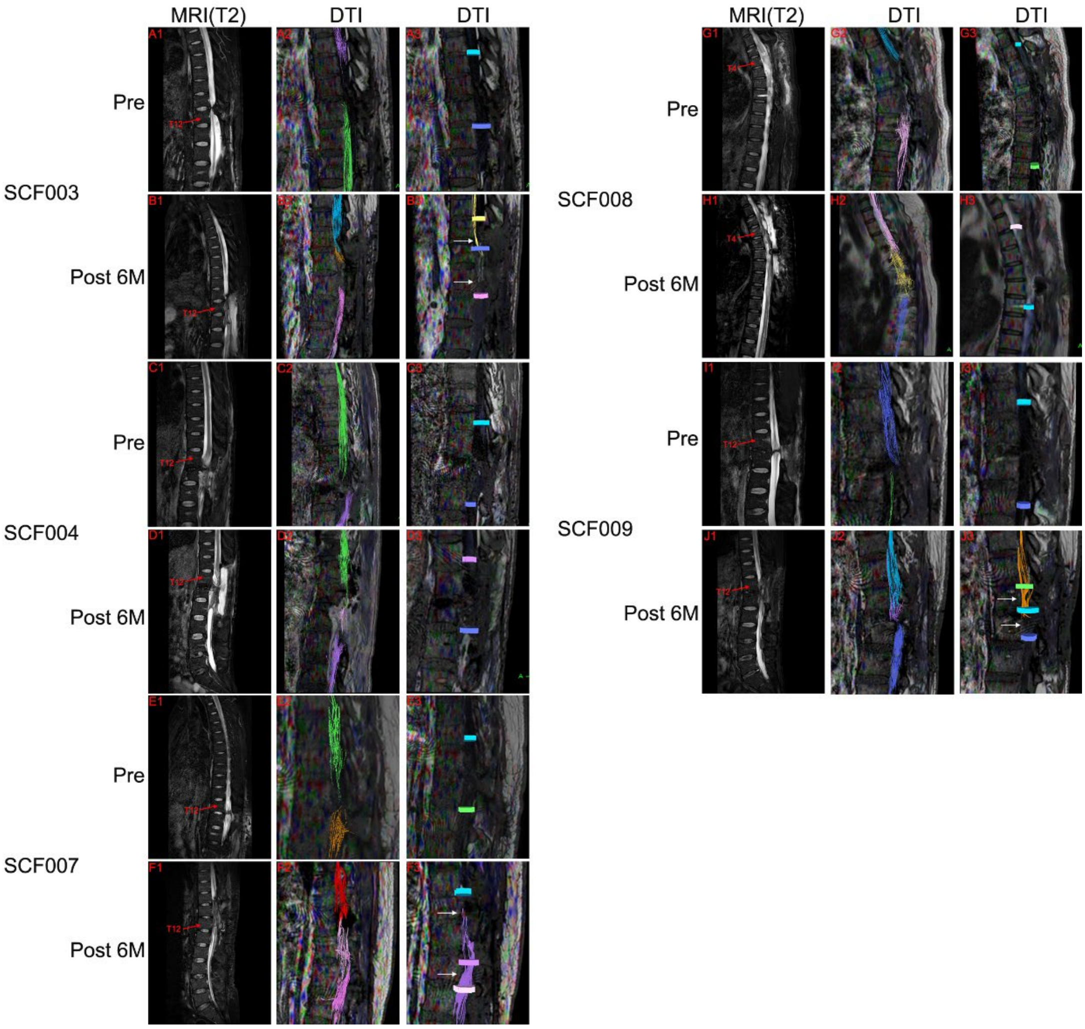

Fig.S2 Neuroimages of patients SCF003, SCF004, SCF007, SCF008, and SCF009. Preoperative T2-weighted MRI scans show spinal cord injury (A1, C1, E1, G1, I1), and DTIs show complete disruption of the spinal cord fibers (A2, A3, C2, C3, E2, E3, G2, G3, I2, I3). Postoperative T2-weighted MRI scans show the autotransplanted sural nerve in the original spinal cord injury area (B1, D1, F1, H1, J1). DTI of SCF003 and SCF009 show that the nerve fibers had been reconnected at the site of proximal transection, not at the site of distal transection (white arrow in B3, J3). DTI of SCF007 shows that the nerve fibers had been reconnected at the site of distal transection, not at the site of proximal transection (white arrows in F3). DTIs of SCF004 and SCF008 show no neural connection of nerve fibers at both sites of transection (white arrows in D3, H3). The colors of the fibers were automatically generated by the DTI system of MRI, with no practical significance. Pre: Preoperatively; Post 6M: 6 months postoperatively.

Note: Due to the absorbable rivet factors mentioned in the discussion, DTI of neural connection at the sites of transection were obscured in some patients. These patients will be re-examined for DTI. In addition, because DTI cannot image gray matter, neural continuity of the grey matter across the sites of transection could not be imaged. We are also searching for non-invasive examination methods to image gray matter nerve fibers.
